# Mycetoma

**DOI:** 10.1371/journal.pntd.0014291

**Published:** 2026-05-15

**Authors:** Max Hoekstra, Kirby R. Lattwein, Wendy W. J. van de Sande

**Affiliations:** Department of Medical Microbiology & Infectious Diseases, ErasmusMC, Rotterdam, The Netherlands; Institute of Continuing Medical Education of Ioannina, GREECE

## Abstract

The Neglected Tropical Disease mycetoma is a chronic, progressive infectious disease characterized by large tumor-like masses in subcutaneous tissues. It is endemic in rural areas of low- and middle-income countries, where it can lead to extensive health and socioeconomic burdens for the patient and their family. It is especially prevalent among male farmers. The disease can be caused by both bacteria and fungi, referred to as actinomycetoma and eumycetoma, respectively. Inoculation occurs when a skin breach allows the pathogen to enter the subcutaneous tissue. The pathogen can form grains, which make the disease more resistant to host defense and antimicrobial treatments. This review summarizes the current knowledge on the historical background and epidemiology of mycetoma, the characteristics of its causative agents, underlying pathophysiology, diagnostic approaches, available treatment strategies, preventive measures, and future prospects for disease management and research. Although the recognition of mycetoma as both a neglected tropical disease and a fungal priority pathogen has increased awareness and research interest, large knowledge gaps remain.

## Author summary

Mycetoma is a progressively debilitating infectious disease characterized by large tumor-like lesions, most commonly affecting the feet. It often leads to severe disability if left untreated. People living in rural environments of tropical and subtropical regions are at greatest risk of developing this disease. Mycetoma has profound physical, social, and economic consequences for affected persons and their communities. It can be caused by bacteria or fungi. Accurate diagnosis of the infecting microbe is difficult in resource-limited regions, as reliable diagnosis requires well-equipped laboratories that are often unavailable. Treatment is particularly inadequate for fungal mycetoma, as treatment durations are long, require invasive surgery, and are likely to recur. In this review, we highlight knowledge gaps as we explore the history of mycetoma, why and where it occurs, the organisms that cause it, how it develops in the body, and the current approaches to diagnosis and treatment. We also discuss preventative strategies and future research directions. Finally, we urgently call for better awareness, diagnostic tools that can be used without laboratory infrastructure, and improved treatments to reduce the global burden of mycetoma.

## Introduction

Mycetoma is a neglected tropical disease (NTD), recognized as such by the World Health Organization (WHO) in 2016 [1]. It is characterized by large, tumor-like masses in subcutaneous tissues, most often in the extremities [2]. This disabling disease occurs globally, but predominantly affects rural regions in low- and middle-income countries with a (sub)tropical climate [3,4]. Mycetoma can be caused by either bacterial or fungal pathogens [5]. Bacterial mycetoma is referred to as actinomycetoma, while fungal mycetoma is known as eumycetoma. In 2022, the eumycetoma-causing fungi were added as high-priority pathogens to the WHO fungal priority list [6]. Although the recognition of mycetoma as both an NTD and a fungal priority pathogen has increased awareness and research interest, large knowledge gaps remain, current treatment options are unsatisfactory, and point-of-care diagnostic tests are lacking [7,8].

In this review, we discuss the historical background and epidemiology of mycetoma, the characteristics of its causative agents, underlying pathophysiology, diagnostic approaches, available treatment strategies, preventive measures, and future prospects for disease management and research.

## History

Mycetoma was described as early as 2000–1000 BC in the *Atharva-Veda*, a sacred text from India’s Vedic culture, as *padaavalmika* or “anthill foot” [9,10]. Since then, the disease has been known by multiple names throughout history. From the early 19th century, terms such as *slipatham,* meaning “elephant foot”, were used in the same region, indicating common recognition of the disease [9].

The first modern clinical descriptions of mycetoma appeared in Indian Army Reports in the 1840s, where it was referred to as *Madura* *foot*, after the Madurai region of India [11,12]. In 1874, the physician Henry Vandyke Carter introduced the term *mycetoma* and was the first to postulate its mycotic nature [11,13]. He provided the first mycetoma fungal samples for description by M.J. Berkeley and later submitted black-grained specimens to E. Brumpt, who taxonomized it as *Madurella mycetomatis* in 1894 [10]. Around this same time, the distinction between bacterial and fungal mycetoma became clear, following H. Vincent’s taxonomic description of actinomycetes [10,11].

The first modern description of actinomycetoma was published in 1912 and was a case from Mexico [9]. However, archaeological evidence suggests a much longer history of the disease in that region. Mycetoma-like lesions have been identified in a skeleton belonging to the Tlatilco culture, dating between 1300 and 1000 BC [14]. The remains show characteristic honeycomb-pattern osteolytic and regenerative lesions in the right lower limb. Furthermore, the individual fits modern risk profiles: a male aged approximately 25–30 years, living in a region with a tropical climate, and belonging to a culture that gathered wood and food barefoot [14].

## Epidemiology

The first approximation of mycetoma disease burden was made in 2013 by dividing the number of reported cases by the population of the country at that moment in time [15]. This resulted in a gross underestimation of disease burden, with a reported prevalence of 1.8 per 100,000 inhabitants in Sudan [15]. It is therefore unsurprising that when true prevalence was subsequently determined in endemic Sudanese villages, a 300- to 2000-fold higher prevalence was found, ranging from 6.2 to 35 cases per 1000 inhabitants [16,17].

**Fig 1:**
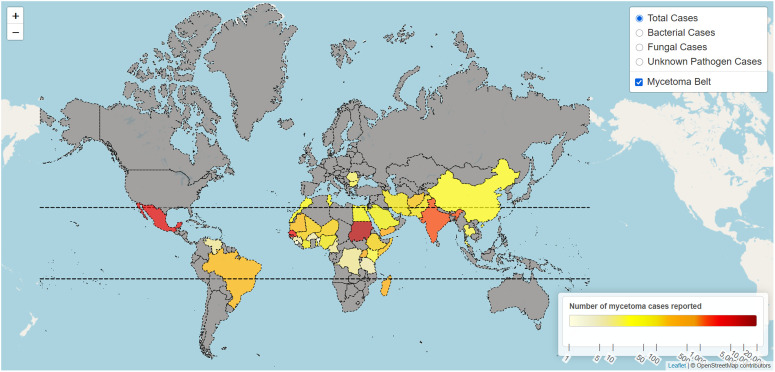
Global distribution of reported mycetoma cases (interactive map). Total reported mycetoma cases per the dataset compiled by Van de Sande and Fahal in 2024 [18]. The interactive **HTML** version of the map allows visualization of all cases, or only eumycetoma or actinomycetoma cases (S1 Fig). There, hovering over a country displays its name, its total number of reported cases, and the number attributed to fungal and bacterial etiologies. The map was made using R v4.3.1 with the leaflet package (version 2.2.2) [24]. The underlying map is OpenStreetMaps and the polygons are based on NaturalEarthData.

Globally, mycetoma is most prevalent in countries located between the latitudes 15°S and 30°N. This area is often referred to as the “mycetoma belt” (Fig 1). These countries are characterized by a (sub)tropical climate, an annual rainfall between 50 and 1000 mm, and mean temperatures in the coldest quarter ranging from 18 to 25 °C [3,18–21]. In the Global North, primary infections are rare, with most cases being imported from endemic areas. Distinct geographical clustering of specific etiological agents has also been observed at a more local scale. For example, in the Senegal-Mauritania region, three ecological zones based on annual rainfall have been identified: areas with 50–250 mm rainfall are predominantly associated with actinomycetoma, 250–500 mm with common eumycetoma species, and 500–800 mm with specific red-grain actinomycetoma and white-grain fungal species [19]. Similar zone-pathogen associations were further confirmed in subsequent Senegalese studies [22,23].

Within these ecological zones, marked differences in prevalence have been observed [15]. In general, mycetoma occurrence is associated with environmental factors such as aridity, proximity to water, small diurnal temperature variations, low soil cation concentrations, wetness index, and the presence of thorny tree species [21,25,26]. For the 1470 actinomycetoma cases studied in Sudan, the strongest predictors were aridity and distance to the nearest lake or pond, followed by distance to the nearest river and mean temperature during the coldest quarter of the year [21,25]. In contrast, eumycetoma prevalence in Sudan was most strongly predicted by distance to the nearest river, followed by daily temperature variation and the presence of *Acacia mellifera* and *Faidherbia albida* trees in the area [21,25]. Patient residences are most commonly located on clay-rich soil, which may contribute to disease risk due to slow water permeation leading to water pool formation [17]. In highly endemic areas, DNA from common eumycetoma causative agents has been identified in environmental samples, including dung, soil, thorns, spines, and walls, although successful culture from these sources has not been achieved [18,27].

## Description of the pathogen

There are more than 80 known causative agents of mycetoma, of which 69 are fungi [5,18]. Given the distinct environmental preferences of these species, the prevalence of individual pathogens varies by geographical region (Table 1). Globally, the most common eumycetoma agent is *Madurella mycetomatis,* followed by *Falciformispora senegalensis*, *Trematosphaeria grisea*, *Scedosporium boydii*, and *Medicopsis romeroi* [18]. The most common actinomycetoma agents are *Actinomadura pelletieri*, *Actinomadura madurae*, *Streptomyces somaliensis*, *Nocardia brasiliensis*, and *Nocardia asteroides* [3].

**Table 1:** Common mycetoma causative agents. Overview of the 5 most common actinomycetoma and eumycetoma causative agents. The table specifies whether the species causes actinomycetoma (bacterial) or eumycetoma (fungal), the typical grain color and approximate size (in millimeters), and whether the organism is the predominant causative agent of actino- or eumycetoma in each world region. An asterisk (*) indicates that the genus is specified, but the species is not.

	Species	Grain color	Grain size (mm)	Major geographical areas	References
Actinomycetoma	*Nocardia brasiliensis*	White	<1.0	Middle and East Africa*; North America; Latin America and the Caribbean; South America	[3,28–32]
	*Streptomyces somaliensis*	Yellow/ Brown	0.5–2.0	West Asia	[3,28–30]
	*Actinomadura madurae*	White/ Yellow/ Pink	1.0–5.0	North Africa; Latin America	[3,28–31,33]
	*Actinomadura pelletieri*	Red	0.3–0.5	West Africa	[3,28,30,34]
	*Nocardia asteroides*	White	<1.0	Middle and East Africa*; South East Asia	[3,28,30–32]
Eumycetoma	*Madurella mycetomatis*	Black	Up to 5.0 or more	North, West and East Africa; South, South East and West Asia	[3,18,28,29,35]
	*Falciformispora senegalensis*	Black	0.5–2.0	West Africa	[3,18,28,35,36]
	*Trematosphaeria grisea*	Black	0.3–0.6	Latin America and the Caribbean; South America	[3,18,28,35]
	*Scedosporium boydii*	White/ White yellow	0.2–2.0	North America*; Middle Africa	[3,18,28,35]
	*Medicopsis romeroi*	Black	0.5–1.5	India	[3,18,28,35,37]

In actinomycetoma, a broad range of grain colors is observed [28,30]. *A. pelletieri* is particularly morphologically distinct due to its red grain formation. Other actinomycetoma agents produce grains ranging in color from pale shades such as white, yellow, or pink to darker brown shades [28,30]. Among the common actinomycetes, *A. madurae* forms the largest grains, typically measuring 1–5 mm in diameter, [29] whereas the red-grained *A. pelletieri* produces much smaller grains, generally ranging from 0.3 to 0.5 mm [34]. *N. brasiliensis* is the most prevalent, especially in highly endemic actinomycetoma regions of Latin and South America [3].

By far the most common eumycetoma causative fungus is *M. mycetomatis*, responsible for approximately 86.0% of cases [18]. Like most other common eumycetoma agents, it forms black grains; a notable exception is *S. boydii,* which forms white to yellow-white grains (Table 1) [18]. *M. mycetomatis* forms the largest grains among the common eumycetoma agents, with grain diameters sometimes exceeding 5 mm. In contrast, *T. grisea* lies on the other end of the spectrum and is the only common eumycetoma agent whose grains do not regularly exceed 1 mm diameter, typically ranging from ~0.3 to 0.6 mm (Table 1) [18,29]. *M. mycetomatis* is the predominant eumycetoma causative agent across most regions of Asia and Africa [3]. In Middle Africa and North America, *Scedosporium* species are most common [3]. In Latin America, *T. grisea* is most commonly reported, but molecular identification studies have indicated that many of these cases were in fact another species and thus misdiagnosed [3,38,39].

## Pathophysiology

Mycetoma is predominantly observed in males [15,40]. This has often been attributed to differences in occupational exposure, however this alone may not fully explain the sex disparity, and hormonal factors have also been suggested to contribute [19,40,41]. Most cases occur in individuals aged 20–50 years, with farmers being the most affected group, whereas individuals in professional or government occupations are less frequently affected [40]. Hospital-based data likely overrepresent severe disease, as patients with early or slowly progressive lesions rarely seek medical care [19]. Host genetic susceptibility is considered an additional risk factor, particularly involving genes related to innate and adaptive immunity, enzymes implicated in grain formation, and sex hormone biosynthesis [41].

Clinically, mycetoma typically begins as small, painless nodules that gradually enlarge, sometimes exceeding 10 cm and forming tumor-like masses [42]. Disease progression varies widely, ranging from weeks to several years, depending on the causative organism and host-related factors [20,43]. Lesions are generally firm and rounded but may also be soft, lobulated, or cystic, and are often mobile [44]. As the disease progresses, sinus tracts develop and discharge pus and grains, the color of which varies according to the causative organism [44]. Pain is uncommon in lesions smaller than 5 cm unless a secondary bacterial infection is present. Secondary bacterial infections are reported in ~18–20% of cases [42,44].

The lower limb is affected in ~76.9% of cases. This is followed by the hand (9.4%), with occasional involvement of the trunk, face, neck, buttock, and groin [19,40]. Infections outside the extremities are more difficult to treat and are associated with increased mortality [18].

Inoculation of the pathogen through disruption of the skin barrier is believed to be necessary for infection. Skin trauma caused by thorns has been strongly associated with mycetoma development, and plant residues have been identified within grains [19,21]. Other causes of skin breaches, including road accidents and animal bites, have also been implicated in pathogen transmission to the subcutaneous tissue [19]. Given this mode of transmission, it is unsurprising that the foot is the most common site of infection, and walking barefoot or with open footwear is considered a major risk factor [16,20,45]. Especially given that the soil is likely to form an environmental reservoir for the pathological agents [18,27]. Although morphological differences in grains are observed depending on the causative organism, no consistent differences in lesion progression or bone involvement have been identified between etiological agents [18].

During disease progression, the infection typically remains localized to the subcutaneous tissue but may spread via lymphatic or hematogenous routes in advanced stages [2]. Furthermore, bone involvement can occur as a result of chronic compression by expanding soft-tissue lesions, causing irritation of the bone surface and pain, followed by deeper invasion into the bone. This allows the pathogen to form cavities within bone and spread further into adjacent structures [42,46].

A defining feature of mycetoma is the formation of grains: dense aggregates of fungal hyphae or bacterial filaments embedded within a cement-like matrix [47]. These grains range from microscopic to approximately 2 mm in diameter [48]. and consist predominantly of host-derived proteins and DNA, accounting for roughly 99.3% of their composition, with only a small fraction originating from the pathogen itself [49,50]. For black grain eumycetoma, melanin is produced and distributed both extracellularly and intracellularly, providing protection against oxidative stress and binding antimicrobial compounds [7,51].

Grain formation has been reproduced in both mammalian and invertebrate models. In mice, grain models have been established for the actinomycetoma agent *N. brasiliensis,* and the eumycetoma agent *M. mycetomatis* [52,53]. In the invertebrate *Galleria mellonella,* grains have been formed for *A. madurae, M. mycetomatis,* and *F. senegalensis* [47,54,55]. Detailed histological, proteomic, and transcriptomic analyses of *M. mycetomatis* grain formation in *G. mellonella* revealed a multistep process*.* In this invertebrate, infection starts with recognition of loose fungal hyphae by the pattern recognition receptors of the host, leading to immune cell aggregation around the hyphae [50]. The fungus subsequently produces cement material, facilitating cross-linking between pathogen and host. Following this, the fungus releases zincophores and siderophores to acquire zinc and iron, allowing it to also cross-link and further stabilize the extracellular matrix [50,56]. Host cells produce reactive oxygen species and antimicrobial peptides, while *M. mycetomatis* undergoes melanization as a protective response [50,57,58]. Ultimately, host cell breakdown occurs within the grain, which becomes fully melanized and encapsulated by collagen [50,57]. The mature grain is then surrounded by large amounts of host immune cells, resulting in granuloma formation.

## Diagnosis

Accurate and early diagnosis of mycetoma is imperative for achieving favorable treatment outcomes. Diagnosis relies on a combination of clinical assessment, imaging techniques, and laboratory methods to correctly identify both the causative organism and the extent of disease [8,48]. Most diagnostic approaches require a well-equipped laboratory and are invasive, time-consuming, and costly, which poses significant challenges in endemic regions [51].

**Fig 2:**
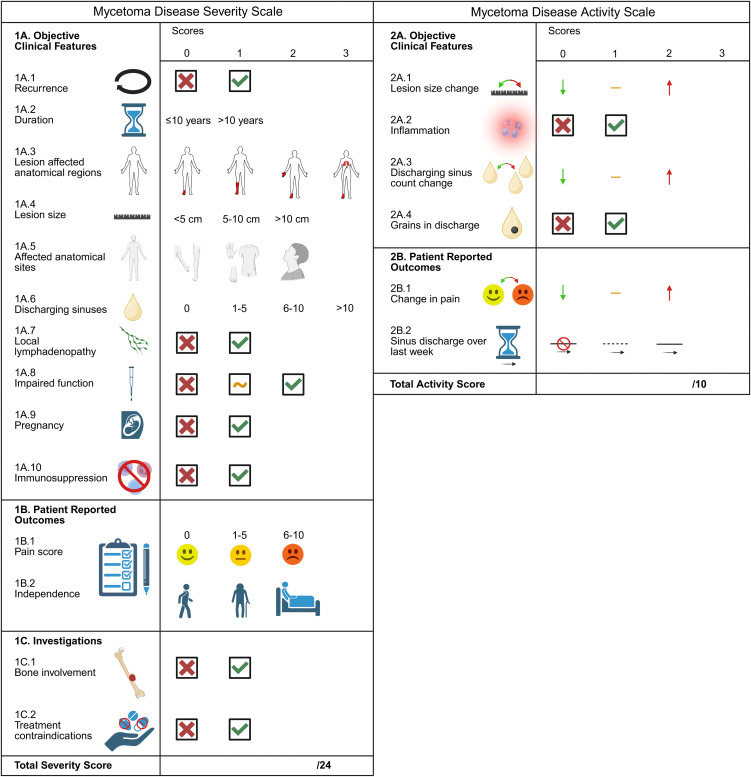
Mycetoma Activity and Severity Clinical Scale infographic. Column 1 depicts the mycetoma disease severity scale, and column 2 depicts the mycetoma disease activity scale. Each item is scored from 0 to 3 points. Severity and activity scores are recorded at initial patient presentation and at all follow-up appointments. Scores are expressed as X out of 24 points for disease severity and X out of 10 points for disease activity, with higher scores indicating more severe or active disease. Within column 1, section 1A shows objective clinical features, 1B patient reported outcomes, and 1C findings from additional investigations. A condensed explanation of the individual items is provided as follows: 1A.1: Reappearance of disease at the same site within 3 months. 1A.2: Disease duration. 1A.3: Lesion extent and localization, score 0 • single lesion confined to one anatomical site; score 1 • single lesion spanning two adjacent anatomical sites; score 2 • two or more lesions in different anatomical sites; score 3 • spread of lesion into internal organs or body cavities. 1A.4: Largest lesion diameter (cm) measured from the surface. 1A.5: Anatomical sites involved; score 0 • arms or legs; score 1 • hands, feet, torso, or pelvic region; score 2 • head or neck. 1A.6: Number of open or discharging sinus tracts. 1A.7: Local lympodenopathy, defined as infection of the lymph nodes draining the infection site. 1A.8: Impact on function of the affected joint or limb (not relevant if no joint or limb is affected), score 0 • no impact or not relevant; score 1 • impaired function and/or movement; score 2 • complete loss of function or movement. 1A.9: Pregnancy. 1A.10: Immunosuppression. 1B.1: Patient-reported pain severity using a visual pain scale from 0 (no pain) to 10 (worst imaginable pain), score 0 • absent to mild pain graded 0-3; score 1 • moderate pain, graded 4-6; score 2 • severe pain, graded 7-10. 1B.2: Ability to perform daily living activities independently, score 0 • independent; score 1 • independent with difficulty; score 2 • dependent on others. 1C.1: Evidence of bone involvement on imaging (X-ray, CT or MRI). 1C.2: Contraindications to certain drug regimens, (e.g., abnormal liver function, drug interactions, renal insufficiency, adrenal insufficiency, or anemia). 2A.1: Change in mycetoma size of ≥10%, score 0 • reduction; score 1 • no change larger than 10%; score 2 • enlargement. 2A.2: Active inflammation, present if the skin overlying the lesion feels warmer compared to surrounding skin and/or increased sweating at the lesion site. 2A.3: Change in the number of open or discharging sinus tracts, score 0 • reduction or all closed; score 1 • no change or first assessment; score 2 • increase. 2A.4: Presence of grains in sinus tract discharge. 2B.1: Change in pain severity. 2B.2: Frequency of sinus tract discharge over the last week, score 0 • no discharge; score 1 • intermittent discharge; score 2 • continuous discharge. This infographic was developed based on the Mycetoma Activity and Severity Scale (MASS) [59]. Created in BioRender. Hoekstra, M. (2026) https://BioRender.com/d8uzwr9.

Initial identification generally starts with clinical evaluation of the lesions and examination of discharged grains. Grain size, color, and consistency vary by causative agent, providing a starting point for identification (Table 1) [48]. The Mycetoma Activity and Severity clinical Scale (MASS) was developed based on clinical expert consensus in a Delphi study (Fig 2) [59]. This scale provides a structured framework for assessing mycetoma disease severity and activity and is intended for use as a standardized assessment tool in clinical practice, clinical research, and endemic care settings.

Imaging techniques such as X-ray, ultrasound, CT, and MRI are crucial for assessing disease extent [51]. Using MRI, mycetoma can be identified by hypointense lesions with hyperintense centers within them, giving a characteristic “dot-in-circle” appearance [60]. Ultrasound, which is more feasible in low-resource settings, and indicate whether the causative agent is fungal or bacterial based on grain echogenicity patterns. With ultrasound thick cavity walls are often associated with eumycetoma, while more compact grain aggregations are more common for actinomycetoma [61,62]. The technique cannot identify the causative agent at the species level.

Direct microscopic examination of grains using 10% potassium hydroxide provides a fast and inexpensive presumptive diagnosis [48,63]. This technique digests mucus and keratin, creating a clear background for observation and facilitating diagnosis based on grain color and size, which can be suggestive of *Nocardia*, *Actinomadura*, or eumycotic species. However, it lacks specificity and cannot reliably distinguish between all causative species [28,48]. Gram, Ziehl-Neelsen, modified Kinyoun, periodic acid-Schiff, and Grocott staining are staining techniques that can be used to differentiate between bacterial and fungal causality [48,64].

Fine-needle aspiration cytology (FNAC) and imprint smears are simple, inexpensive methods that can provide rapid diagnosis [65,66]. FNAC involves aspirating material from the lesion under aseptic conditions and preparing cell blocks for staining [65,66]. Performing FNAC under ultrasound guidance reduces false-negative results, with a sensitivity of 97.1% and specificity of 100% for actinomycetoma, and both a sensitivity and specificity of 100% for eumycetoma [67]. These results are comparable to those obtained with histopathology, which involves invasive deep surgical biopsies followed by time-consuming examination for characteristic tissue reactions to the causative organisms [65,68].

Culture remains a standard method for definitive identification, but it is slow, labor-intensive, and has low accuracy [28,48]. Multiple grains are required, which are washed and inoculated onto various media at different temperatures, with and without antibiotics [48]. The temperature and media associated with growth combined with colony morphology and fruiting body morphology are then used to identify the causative agent [48,51].

Serological assays, including ELISA, immunodiffusion, and immunoblotting, have been explored, however their clinical utility remains limited due to cross-reactivity, lack of standardization, and poor specificity [28,48,51]. Serological diagnosis is used in a limited number of centers in Mexico and can be useful for the identification of *N. brasiliensis* [31].

Molecular identification methods currently have the highest specificity for the identification of mycetoma causative agents [48]. Molecular approaches primarily rely on nucleic acid amplification and sequencing techniques, including PCR, 16S rRNA sequencing, rolling circle amplification (RCA), loop-mediated isothermal amplification (LAMP), and recombinase polymerase amplification (RPA) [69–72]. While these techniques all offer excellent specificity and sensitivity, their widespread use is limited by their high cost and infrastructure requirements [8].

In addition to utilizing amplification-based strategies, high-throughput proteomic techniques using matrix-assisted laser desorption/ionization-time-of-flight mass spectrometry (MALDI-TOF MS) enable rapid and accurate identification of etiological agents [39]. Standard MALDI-TOF cannot reliably identify *Nocardia* because of its mycolic-acid rich cell wall, instead requiring ethanol-formic acid extraction [73]. Although MALDI-TOF MS has a high up-front cost, it is less labor-intensive, faster, and more cost-effective than sequencing-based molecular identification methods [74]. Molecular identification methods are generally superior to culture-based methods due to their high speed and accuracy, direct usage of clinical samples, and lower labor intensity, however, their application in endemic settings remains limited due to resource constraints [8,28].

Currently, no point-of-care diagnostic test is available for mycetoma. This limited diagnostic capacity leads to misdiagnosis and delays in appropriate therapy, allowing disease progression before effective treatment is started and leading to poorer outcomes.

## Treatment

Treatment strategies differ substantially between actinomycetoma and eumycetoma. Actinomycetoma is primarily treated with a combination of different antimicrobials over months to >1 year, supplemented by surgery when necessary. In Mexico, treatment with trimethoprim-sulphamethoxazole and amikacin sulfate, known as the Welsh regimen, is primarily used for recalcitrant lesions or those located near vital organs [75]. In contrast, in Sudan where actinomycetoma is mainly caused by *Actinomadura* and *Streptomyces* species, first-line treatment consists of trimethoprim-sulfamethoxazole combined with amoxicillin-clavulanic acid [76,77]. In case of treatment failure, it was recently demonstrated that combining trimethoprim-sulfamethoxazole with linezolid resulted in favorable outcomes for Nocardia species and *A. madurae,* especially in cases where regular treatment failed [78,79]. Due to the prolonged treatment durations and high dosages, these regimens are associated with an increased risk of adverse effects [80]. Despite these issues, actinomycetoma treatment achieves medical cure rates of up to 90% of patients [81].

The most commonly used treatment for eumycetoma consists of 6 months of oral itraconazole given twice-daily, followed by surgical removal of the lesion and continuation of itraconazole for at least six more months postoperatively to reduce the risk of recurrence [82]. According to a WHO survey, itraconazole was used by 85% of respondents [83]. Next to itraconazole, terbinafine (58%), voriconazole (41%), posaconazole (33%), ketoconazole (30%), and injectable amphotericin B (36%) were also reported to be used [83]. Itraconazole bioavailability varies based on formulation and food intake [84]. It is metabolized by the liver via CYP3A4 into its active metabolite hydroxyitraconazole, with respective half-lives of ~24 and 12 hours. The drug is highly lipophilic, exhibits strong protein binding (>99%), and has a broad tissue distribution [85,86]. Its antifungal activity primarily works by inhibiting lanosterol 14α-demethylase (CYP51A), which reduces ergosterol production leading to fungal cell membrane disruption [85–88].

In the first and, to date, only clinical trial for eumycetoma, itraconazole combined with surgery achieved an 80% cure rate in patients with relatively small lesions and no bone involvement [89]. In daily practice, reported cure rates are lower, with a corrected cure rate of 67.6% [18]. These lower success rates are the result of the causative organism, stage of infection, lesion size, bone involvement, presence of secondary bacterial infections, and uncommon anatomical locations such as the trunk, neck, or head [18,90]. Furthermore, treatment is routinely discontinued in endemic settings. At the Sudanese Mycetoma Research Center, 54% of patients did not complete treatment, partly due to limited financial resources and drug availability, but also due to low trust in and social support facilitated by biomedical care [83,91,92].

Importantly, itraconazole monotherapy during the first six months did not result in reductions in pain, swelling, sinus formation and discharge, or (1–3)-β-D-glucan serum levels [89,93]. Only after surgery clinical improvement was observed, indicating that surgery is essential for curative treatment. Surgerical approaches range from local excision for lesions smaller than 10 cm, to debulking procedures or amputation in advanced disease [94].

Besides azoles, oral terbinafine administered at 1000 mg/day has shown efficacy and is well-tolerated as an antifungal agent for eumycetoma. In an open-label clinical study without surgery involving 20 patients in Senegal, terbinafine treatment resulted in clinical improvement in 80% of patients infected with *M. mycetomatis* or *F. senegalensis* [95]. A retrospective Senegalese study reported a cure rate of 55.6% [22,95].

Fosravuconazole, a prodrug of ravuconazole with favorable pharmacokinetics, is a promising alternative antifungal agent for eumycetoma [84]. Ravuconazole has demonstrated potent *in vitro* activity against a broad range of causative agents and efficacy in *G. mellonella* infection models of *M. mycetomatis* [96,97]. Fosravuconazole was evaluated in a first randomized clinical trial for *M. mycetomatis* mycetoma, administered once weekly at doses of 200 mg or 300 mg [89]. The 50% higher dose resulted in 75% higher serum levels, but was not associated with better outcomes [84]. Drug exposure exceeded MIC90 values throughout the 12-month study, and a cure rate of 85% was achieved with the 200 mg dose combined with surgery [84,89]. Although fosravuconazole efficacy was not superior to the standard itraconazole regimen, it offers practical advantages, including once-weekly dosing due to its long half-life, low potential for drug-drug interactions, and good tolerability may improve treatment adherence and cost-effectiveness [84,89].

Despite the availability of multiple antifungal agents, treatment of eumycetoma remains challenging. It is mainly managed by the azoles, terbinafine, and injectable amphotericin B. Itraconazole is an effective treatment for the majority of the eumycetoma causative agents. However, in the Sudanese participants of the *M. mycetomatis* clinical trial, measured serum concentrations of itraconazole plus hydroxyitraconazole were much lower than expected and consistently below the MIC90 for *M. mycetomatis* (MIC90 = 0.25 μg/ml, [98]) [18,84]. For other causative agents, including *T. grisea*, *S. boydii*, and *M. romeroi,* MIC90 values exceeded what is achievable *in vivo.* Furthermore, *M*. *fahalii* exhibits intrinsic resistance to itraconazole due to CYP51 mutations [99,100]. *T. grisea* and *M. romeroi* showed improved susceptibility to the newer azoles voriconazole and posaconazole, with MIC90 values within therapeutic serum ranges [18]. However, antifungal activity against hyphae does not necessarily translate to efficacy in more complex systems where grain formation is involved. Murine *M. mycetomatis* grains were reported to have a 100-fold higher MIC for antifungal agents than those observed in hyphae [101].

Drug repurposing initiatives have further identified candidate compounds, including benzimidazoles, 2-aminothiazoles, and fenarimols. These exhibit potent *in vitro* activity and improved survival in *G. galleria* infection models [96,102–104]. Open science initiatives, such as Mycetoma Open Source (MycetOS), offer promising frameworks to accelerate the drug discovery and development pipeline for neglected diseases [105]. Despite available therapies, treatment outcomes remain suboptimal for many patients, highlighting the need for improved preventative strategies and future innovations in diagnosis and care.

## Prevention

No clear prevention guidelines for mycetoma have been established, as no thorough studies on preventative measures have been performed. Because mycetoma is generally accepted to result from skin-penetrating injuries, particularly those caused by with acacia thorns, the prevailing consensus is that wearing sturdy, closed footwear offers an effective protective measure in endemic areas, especially in rural outdoor environments [21]. Similarly, using protective garments like gloves and long trousers during high-risk tasks like threshing, walking in thorny areas, and animal handling, may reduce the risk of skin barrier breaks and subsequent infection.

A One Health strategy may further contribute to mycetoma prevention, by addressing the interconnected roles of humans, animals, and environmental health. This is especially important given the highly suspected reservoir of mycetoma causative agents in soil, plant material, and animals. Utilizing knowledge of environmental risk factors could help reduce direct contact between people and potential reservoirs through the creation of targeted guidelines, for example, on animal handling, waste management, and environmental hygiene. However, current understanding of functional disease reservoir dynamics and transmission pathways in the context of mycetoma causative agents remains limited, restricting the ability to design effective programs to break disease transmission chains.

Evidence supporting community-based preventive strategies is emerging. A cross-sectional study in Sudan utilizing numerous interventions reported reduced recurrence and hospitalization rates, improved early case detection, and higher cure rates following implementation of multiple community-level measures [16]. Interventions employed by this study included free access to medical and surgical treatments; community health education; engagement of community leaders and local artists in advocacy and awareness activities; active early case finding; improved village hygiene; provision of protective footwear; introduction of hygienic animal cages; and passing laws banning animals inside the village. Unfortunately, based on the study design, it was not possible to discern the effectiveness of the individual interventions used or to determine their cost-effectiveness.

The effectiveness of specific preventative interventions is likely dependent on local environmental conditions and sociocultural contexts of the specific endemic situation. Therefore, adaption to local environmental conditions and cultural structures should be considered essential for such preventive measures in other areas. Broadly applicable measures are likely include the use of protective garments during high-risk activities, limiting contact between people and potential reservoirs, active early case detection, and sustained availability of effective pharmaceuticals to prevent disease progression, recurrence, and drug resistance.

## Future prospects

The recognition of mycetoma as a neglected tropical disease by the WHO in 2016, followed by the inclusion of eumycetoma causative agents on the WHO fungal priority pathogen list, has helped raise visibility and interest in the disease. However, mycetoma awareness remains low, even within the infectious diseases research community. A critical first step toward improved control of mycetoma should be to gain a more accurate understanding of the global disease burden, which remains poorly reported.

From a clinical perspective, major gaps persist across prevention, diagnosis, and treatment. So far, little is known about preventative measures that can be taken to reduce mycetoma incidence. Studies should be systematically performed to properly evaluate the effectiveness and cost-effectiveness of high-potential prevention strategies, such as community health education, active early case finding, One Health-based approaches targeting environmental and animal reservoirs, and wearing protective garments during high-risk activities.

Therapeutic development for mycetoma remains limited. Currently, only one randomized clinical trial evaluating two antifungal drugs has been conducted for mycetoma, and most treatment recommendations are derived from small case series. This is specially problematic for eumycetoma, where outcomes remain suboptimal, and treatment duration is long. Future studies should be performed to determine how best to utilize the available treatments by determining their superiority, taking into account differences for causative agents, disease stage, and anatomical extent. Platforms such as CURE ID (https://cure.ncats.io) can support this effort by aggregating real-world clinical data, enabling better characterization of treatment responses across various causative agents and settings [83].

Furthermore, new drug strategies need to be developed to improve our ability to penetrate into or disrupt the mycetoma grain. This could enable shorter, more effective treatments and potentially reduce the need for surgical intervention. This would reduce both the financial and time burden on both patients and healthcare systems, likely resulting in better treatment adherence as well. For this, several *in vitro* and *in vivo* models are currently available. Enhanced penetration of the mycetoma grain could be achieved in various ways, including the development of fungicidal drugs capable of penetrating the grain matrix, chemical modification of existing molecules to improve penetration [102], and the development of targeted drug delivery systems. Alternatively, early inhibition of host or fungal pathways involved in grain formation could have potential in overcoming the challenges posed by grain formation, although such approaches might be less effective in advanced disease.

Diagnostic capacity in endemic settings remains limited by resource-availability, resulting in the usage of suboptimal diagnostic techniques. There is an urgent need for a point-of-care diagnostic test, preferably one molecular in nature, as the absence of such a test will continue to contribute to misdiagnosis and delayed treatment.

Overall, the increased attention generated by WHO recognition should be leveraged to drive coordinated efforts for the creation of more awareness, *in vitro* models, and clinical trials, the development of point-of-care diagnostics, indexation of the global disease burden, validation of prevention strategies, and the development of new drug strategies capable of overcoming grain-associated resistance. All are essential to reduce the global impact of mycetoma.

## Supporting information

S1 Fig Global distribution of reported mycetoma cases (interactive HTML). Total reported mycetoma cases per the dataset compiled by Van de Sande and Fahal in 2024 [18]. This version of the map allows visualization of all cases, or only eumycetoma or actinomycetoma cases. Hovering over a country displays its name, its total number of reported cases, and the number attributed to fungal and bacterial etiologies. The map was made using R v4.3.1 with the leaflet package (version 2.2.2) [24]. The underlying map is OpenStreetMaps and the polygons are based on NaturalEarthData.
(HTML)
